# Barley grain (1,3;1,4)-β-glucan content: effects of transcript and sequence variation in genes encoding the corresponding synthase and endohydrolase enzymes

**DOI:** 10.1038/s41598-019-53798-8

**Published:** 2019-11-21

**Authors:** Guillermo Garcia-Gimenez, Joanne Russell, Matthew K. Aubert, Geoffrey B. Fincher, Rachel A. Burton, Robbie Waugh, Matthew R. Tucker, Kelly Houston

**Affiliations:** 1 The James Hutton Institute, Invergowrie, Dundee, DD2 5DA Scotland, UK; 2 ARC Centre of Excellence in Plant Cell Walls, School of Agriculture, Food and Wine, University of Adelaide, Waite Campus, Glen Osmond, SA 5064 Australia; 3 Plant Sciences Division, College of Life Sciences, University of Dundee. Dundee, DD1 5EH Scotland, UK; 4 Present Address: Guillermo Garcia-Gimenez, Agriculture & Food, Commonwealth Scientific and Industrial Research Organization (CSIRO), Canberra, ACT 2601 Australia

**Keywords:** Genetics, Plant sciences

## Abstract

The composition of plant cell walls is important in determining cereal end uses. Unlike other widely consumed cereal grains barley is comparatively rich in (1,3;1,4)-β-glucan, a source of dietary fibre. Previous work showed *Cellulose synthase-like* genes synthesise (1,3;1,4)-β-glucan in several tissues. *HvCslF6* encodes a grain (1,3;1,4)-β-glucan synthase, whereas the function of *HvCslF9* is unknown. Here, the relationship between mRNA levels of *HvCslF6*, *HvCslF9*, *HvGlbI* (1,3;1,4)-β-glucan endohydrolase, and (1,3;1,4)-β-glucan content was studied in developing grains of four barley cultivars. *HvCslF6* was differentially expressed during mid (8–15 DPA) and late (38 DPA) grain development stages while *HvCslF9* transcript was only clearly detected at 8–10 DPA. A peak of *HvGlbI* expression was detected at 15 DPA. Differences in transcript abundance across the three genes could partially explain variation in grain (1,3;1,4)-β-glucan content in these genotypes. Remarkably narrow sequence variation was found within the *HvCslF6* promoter and coding sequence and does not explain variation in (1,3;1,4)-β-glucan content. Our data emphasise the genotype-dependent accumulation of (1,3;1,4)-β-glucan during barley grain development and a role for the balance between hydrolysis and synthesis in determining (1,3;1,4)-β-glucan content, and suggests that other regulatory sequences or proteins are likely to be involved in this trait in developing grain.

## Introduction

(1,3;1,4)-β-Glucan is a plant cell wall polysaccharide that is found in a range of tissues in barley including the grain, where it constitutes approximately 70% dry weight (w/w)^1^ of endosperm primary cell walls. This polysaccharide accumulates throughout barley endosperm development. The four main phases that define endosperm development in barley and other cereals include the syncytial, cellularization, differentiation and maturation (also referred to as desiccation) phases, each of which are characterized by multiple morphological changes^2,3^. In barley, at 4–5 days after pollination (DAP), (1,3;1,4)-β-glucan is deposited in endosperm cell walls and is uniformly distributed by 10 DAP. (1,3;1,4)-β-Glucan increases in abundance between 16–36 DAP, coinciding with the grain filling and maturation stages^4^. A wide range of natural variation for this trait has been described in contemporary barley cultivars [~2.5–8% w/w;^5,6^] and wild barleys, *Hordeum vulgare* ssp. *spontaneum* [4.5–13.2% w/w^7^]. Barley has considerably higher grain (1,3;1,4)-β-glucan concentration (4–10% w/w) compared to other small grain cereals such as wheat (1% w/w) or rice (<0.06% w/w)^8^. In wheat, the sequence of deposition of cell wall polysaccharides differs from barley, mainly due to the early accumulation of arabinoxylan during endosperm cellularization^9^. While (1,3;1,4)-β-glucan is mainly detected in the central cell walls of wheat endosperm and decreases considerably in late endosperm development stages^9^, an even distribution of (1,3;1,4)-β-glucan abundance has been described in developing barley and *Brachypodium* grain^10^. In *Brachypodium*, the highest levels of (1,3;1,4)-β-glucan are detected during grain filling (17 DAP), showing only a small decrease upon the end of grain desiccation, when the polysaccharide represents up to 45% of the grain^10,11^. Apart from phenotypic differences in grain (1,3;1,4)-β-glucan concentration amongst members of the Poaceae family, it has been shown that the differential expression of the *CslF6* gene, which encodes a (1,3;1,4)-β-glucan synthase^12^, can only partially explain variation in (1,3;1,4)-β-glucan content during grain development^13–15^. Additionally, recent studies suggest that HvCSLF6 has the capability to introduce both (1,3)- and (1,4)-β-linkages within this polysaccharide which generate cellotriosyl (DP3) and cellotetraosyl (DP4) units, affecting (1,3;1,4)-β-glucan solubility and downstream applications^16,17^. Differences in DP3/DP4 ratio are influenced by various environmental and genetic factors and also described across barley cultivars and grain compartments^18^.

During barley endosperm development, *HvCslF6* and *HvCslF9* are the most abundant transcripts of the *HvCslF* gene family. Expression of *HvCslF9* at 3–8 DAP coincides with the initial appearance of (1,3;1,4)-β-glucan in endosperm cell walls^4,13^. However, the function of *HvCslF9* is not entirely clear and it cannot be assumed that all members of the *CslF* gene family encode (1,3;1,4)-β-glucan synthases^19^. By contrast, *HvCslF6* is well characterized as a (1,3;1,4)-β-glucan synthase that is expressed throughout grain development^12^. Variation in *HvCslF6* transcript levels during late grain development coincide with differential accumulation of grain (1,3;1,4)-β-glucan in different barley cultivars^15^. Although it is unclear whether this variation is causative, it provides *prima facie* evidence that variation in *HvCslF6* expression could lead to differences in (1,3;1,4)-β-glucan concentration in mature grain.

In addition to polysaccharide biosynthesis, multiple studies suggest that variation in grain (1,3;1,4)-β-glucan content is also influenced by polysaccharide remodelling and degradation pathways^13,20–22^. Two barley genes, *HvGlbI* and *HvGlbII*, which encode (1,3;1,4)-β-glucan endohydrolases, have been suggested to contribute to this trait, having an impact on malt (1,3;1,4)-β-glucan modification^23–25^. *HvGlbI* is the most abundant (1,3;1,4)-β-glucan endohydrolase expressed during endosperm differentiation, in embryonic tissue (scutellum), roots and during germination^26^, whereas *HvGlbII* is highly expressed in the aleurone of germinated grain, under gibberellic acid regulation^13,26,27^ and simulated malting conditions^28^.

While several naturally occurring polymorphisms have been identified in non-coding regions of *HvCslF6*^29–31^, only three are located within exons and only one affects the protein sequence (A590T). This non-synonymous mutation was identified in the betaglucanless (*bgl*) mutant^29^, where it is associated with reduced (1,3;1,4)-β-glucan content and poor agronomic characteristics. However, as reported in a wide range of barley accessions with phenotypic extremes for this trait^5,30^, the A590T SNP is not diagnostic for grain (1,3;1,4)-β-glucan content. Additionally, Taketa *et al*.^29^ described three Sodium azide-induced alleles of *bgl* which are the result of non-synonymous mutations in highly conserved nucleotides and lead to a lack of (1,3;1,4)-β-glucan.

Two of the end user markets of barley have distinct preferences regarding grain (1,3;1,4)-β-glucan content. Barley cultivars with high (1,3;1,4)-β-glucan content are preferred by the food sector as this polysaccharide is not digested by humans and therefore contributes to total dietary fibre intake, acting against several human health conditions^32–34^. Conversely, the brewing and distilling sector require barley cultivars with low (1,3;1,4)-β-glucan content for an efficient malting process^35,36^. To date *HvCslF6* is the main gene known to functionally contribute to the synthesis of grain (1,3;1,4)-β-glucan content. However, several studies^5,29–31^, have revealed a lack of variation in the *HvCslF6* coding sequence (CDS), suggesting that another aspect of *HvCslF6*, such as the differential regulation of transcript abundance might contribute to variation in the observed phenotype. Previous quantitative trait loci (QTL) studies have suggested that in addition to *HvCslF6*, both *HvCslF9* and *HvGlbI* could contribute to variation in the grain (1,3;1,4)-β-glucan^5,37^. To investigate this further we quantified transcript abundance of all three genes in a grain development series across several barley accessions with divergent grain (1,3;1,4)-β-glucan levels. In the same experiment we quantified (1,3;1,4)-β-glucan levels throughout grain development to assess the temporal deposition pattern of this polysaccharide. In parallel, to investigate sequence diversity of *HvCsl6* in a wider collection of germplasm than previously examined, we used a combination of existing exome capture data and *de novo* sequencing of 3,000 bp of the *HvCslF6* promoter.

## Results

### Variation in grain morphology does not explain (1,3;1,4)-β-glucan content

To identify whether any phenotypic measurements associated with grain morphology could indicate the amount of (1,3;1,4)-β-glucan in mature seeds, seven traits [seed weight, roundness, length, width, thickness, overall area and (1,3;1,4)-β-glucan content] were measured using 153 barley 2-row European elite barley cultivars (Table S1). Correlation coefficients between mature grain (1,3;1,4)-β-glucan content and grain phenotypic traits ranged from −0.006 to 0.108 (Table 1) indicating a lack of correlation between these traits.

**Table 1:** Correlation coefficients of seed weight, roundness, length, width, thickness, overall area and (1,3;1,4)-β-glucan content across 153 2-row elite barley cvs.

Trait	Seed weight	Roundness	Length	Width	Thickness	Seed area	(1,3;1,4)-β-glucan content
Seed weight	—						
Roundness	−0.296	—					
Length	0.266	0.133	—				
Width	0.707	−0.399	0.227	—			
Thickness	0.632	−0.609	0.118	0.566	—		
Seed area	0.661	−0.144	0.501	0.722	0.374	—	
(1,3;1,4)-β-glucan content	0.108	0.013	0.049	0.041	−0.006	0.006	—

The assessment of grain characteristics across 2-row cultivars (cvs): Dew, Imidis, Egmont and Gull used in the grain development series showed significant differences in average TGW, grain surface area and its components of width and length (p-value < 0.01) as shown in Fig. S1. The cvs Imidis and Egmont showed significantly higher average TGW (68.77 ± 0.20 and 59.95 ± 0.58, respectively) and larger grain area (26.57 ± 0.03 and 25.80 ± 0.10, respectively) compared to cvs Dew and Gull (p-value < 0.01, both). Significant differences in grain width (ranged 3.1–3.9 mm) and length (ranged 8.1–9.1 mm) were found across the four cvs (p-value < 0.01, both).

### (1,3;1,4)-β-glucan accumulation is genotype dependent in barley, especially in later stages of grain development

**Figure 1:**
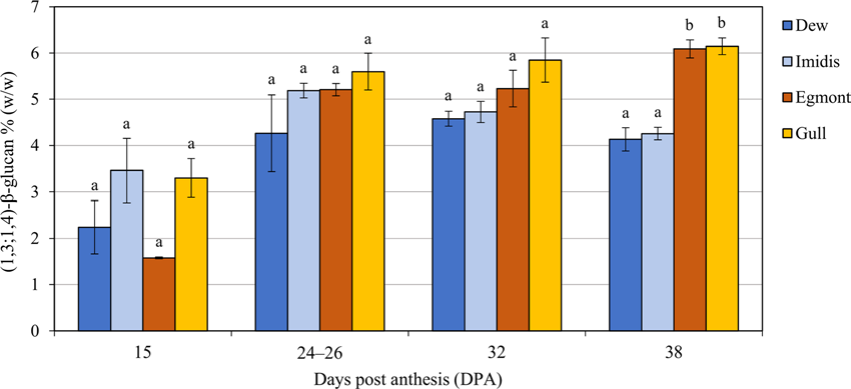
Mean (1,3;1,4)-β-glucan concentration for cvs Dew, Imidis, Egmont, Gull and Morex during grain development stages: 15, 24–26, 32 and 38 days post anthesis (DPA). Bars indicate standard errors from three biological replicates (n = 3). Letters above each bar indicate significant differences within each developmental stage determined by one-way ANOVA followed by Tukey’s test.

To assess changes in grain (1,3;1,4)-β-glucan content over time, the amount of this polysaccharide present in the grain was quantified at two mid (15 and 24–26 DPA) and two late (32 and 38 DPA) grain development stages in cvs Dew, Imidis, Egmont and Gull (Fig. 1). At 15 DPA, (1,3;1,4)-β-glucan was detected in all cvs and ranged from 1.58% (w/w) ± 0.02 to 3.46% (w/w) ± 0.69. A consistent increase in grain (1,3;1,4)-β-glucan levels was observed across all genotypes from 15 until 24–26 DPA, regardless of final (1,3;1,4)-β-glucan concentration in mature grain. Differences between cvs were more obvious towards the end of grain development (32 to 38 DPA) and were only statistically significant at 38 DPA (p-value < 0.01, Tukey’s test). At grain maturity (38 DPA), two main genotype groups were identified based on divergent (1,3;1,4)-β-glucan content, namely cvs Dew and Imidis and cvs Egmont and Gull. In addition, a potential decrease in (1,3;1,4)-β-glucan concentration was observed at 38 DPA compared with the previous grain development time point in 2-row cvs Dew [4.13% (w/w) ± 0.25] and Imidis [4.26% (w/w) ± 0.14]. Conversely, grain (1,3;1,4)-β-glucan concentration increased from 32 to 38 DPA in cvs Egmont [6.09% (w/w) ± 0.19] and Gull [6.15% (w/w) ± 0.18].

### Transcript levels of *HvCslF6*, *HvCslF9* and *HvGlbI* show genotype-dependent and temporal variation during grain development

**Figure 2:**
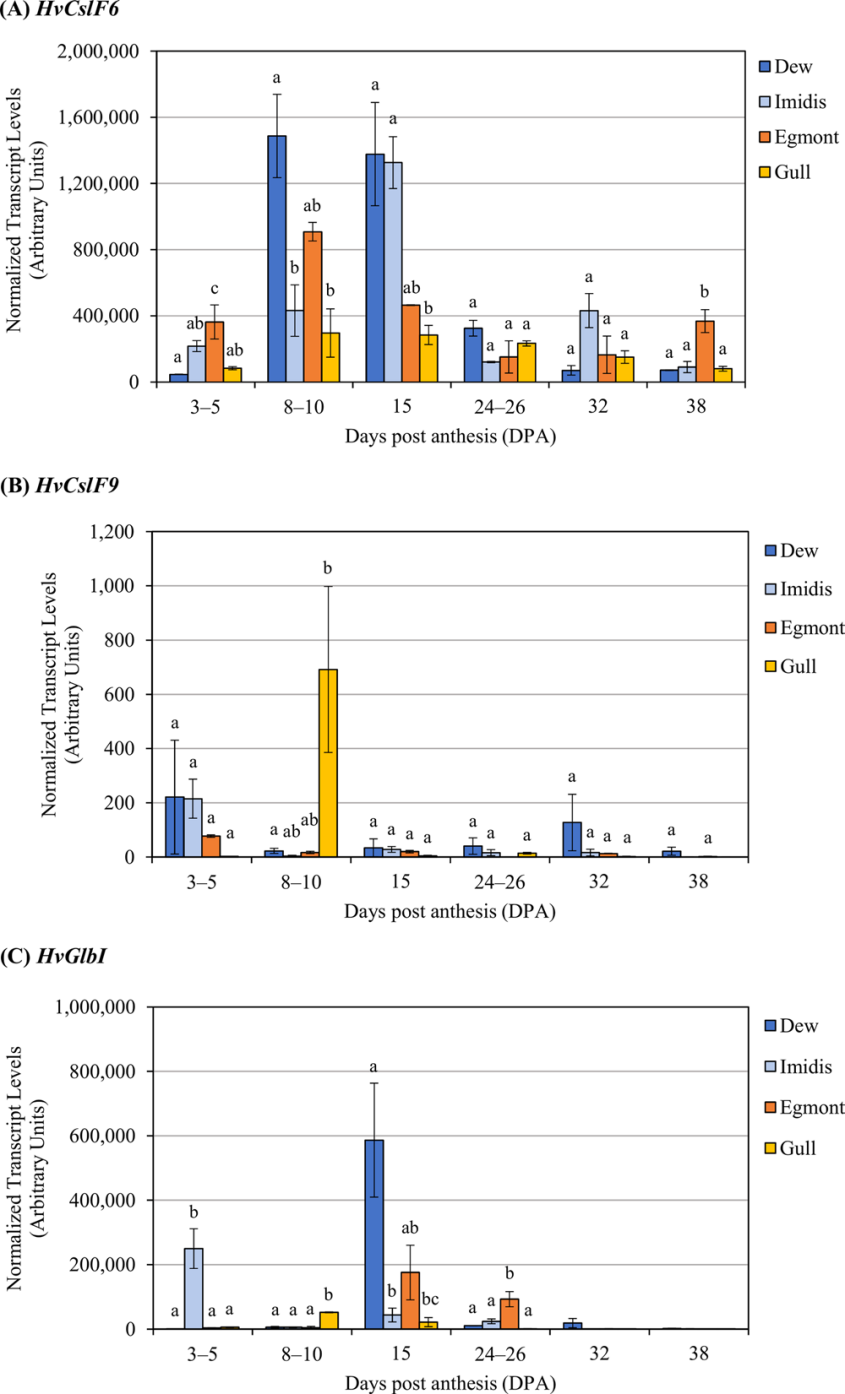
Transcript levels of *HvCslF6*, *HvCslF9* and *HvGlbI* in developing grain of cvs Dew, Imidis, Egmont, Gull and Morex at 3–5, 8–10, 15, 24–26, 32 and 38 days post anthesis (DPA). Transcript levels are expressed as arbitrary units for a) *HvCslF6*, b) *HvCslF9* and c) *HvGlbI*. Expression data was averaged from three biological replicates per genotype and normalized to *α-tubulin*, *GAPDH* and *HSP70* housekeeping genes. qRT-PCR reactions were performed in triplicate. Bars indicate standard errors (n = 3). Letters above each bar indicate significant differences within each developmental stage determined by one-way ANOVA followed by Tukey’s test.

*HvCslF6*, *HvCslF9* and *HvGlbI* transcript abundance were quantified at six stages of grain development: 3–5, 8–10, 15, 24–26, 32 and 38 DPA. As described above, cvs Dew, Imidis, Egmont and Gull show differences in mature grain (1,3;1,4)-β-glucan content, varying from 4.13–6.15% (w/w) (Fig. 1). Variation in *HvCslF6* expression levels was observed between genotypes with similar grain (1,3;1,4)-β-glucan concentration (either high or low) at the same time point (Fig. 2a). *HvCslF6* transcript abundance peaked at 8 DPA in cvs Egmont (high) and Dew (low). The latter showed a prolonged *HvCslF6* expression until 15 DPA. At this time point, higher *HvCslF6* expression levels were found in cvs Imidis (low) and Dew (low) compared to Gull (high; p-value 0.023, Tukey’s test). During mid to late grain development stages (from 15 until 38 DPA), a significant decrease in *HvCslF6* transcript abundance was observed across cvs Imidis (p-value 0.002, Tukey’s test) and Dew (p-value 0.011, Tukey’s test), both with lower grain (1,3;1,4)-β-glucan content compared with cvs Egmont and Gull. Conversely, *HvCslF6* expression levels in cv Egmont (high) showed a moderate increase from 24–26 until 38 DPA (p-value 0.015, Tukey’s test). This expression trend was not observed in cv Gull although it contains a similarly high concentration of (1,3;1,4)-β-glucan in mature grain compared to cv Egmont.

While *HvCslF6* transcript was found at high levels during mid grain development stages (8–10 and 15 DPA), a genotype-dependent response was observed throughout grain development in the present study. In contrast, transcript abundance of *HvCslF9* in all genotypes was extremely low and variable compared to *HvCslF6* (Fig. 2b). *HvCslF9* transcripts were mostly detected during early grain development (3–5 DPA) (cvs Dew, Imidis and Egmont) except in cv Gull where *HvCslF9* expression peaked at 8–10 DPA (p-value 0.037 Tukey’s test). From 15 to 38 DPA, *HvCslF9* transcript abundance remained undetectable for most genotypes except for cv Dew which showed a relative increase towards late stages of grain development, although levels were very low compared to early grain development (3–5 DPA).

The transcript profile of *HvGlbI*, which encodes (1,3;1,4)-glucanase isoenzyme EI, was also determined in this experiment (Fig. 2c). The expression of *HvGlbI* at 15–26 DPA coincides with the differentiation stage of barley grain development. *HvGlbI* transcript abundance was higher in the low grain (1,3;1,4)-β-glucan cvs Dew (at 15 DPA, p-value 0.016, Tukey’s test) and Imidis (at 3–5 DPA, p-value 0.011, Tukey’s test) compared to cvs Egmont and Gull, both with high grain (1,3;1,4)-β-glucan through grain development. For the two cvs with lower levels of grain (1,3;1,4)-β-glucan, Imidis and Dew, we observed a temporal difference in the peak of *HvGlbI* expression, at 3–5 DPA and 15 DPA respectively. *HvGlbI* expression was undetectable towards late grain development stages (32 and 38 DPA).

### *HvCslF6* has low levels of genetic variation in coding and non-coding regions within a diverse collection of barley

**Figure 3:**
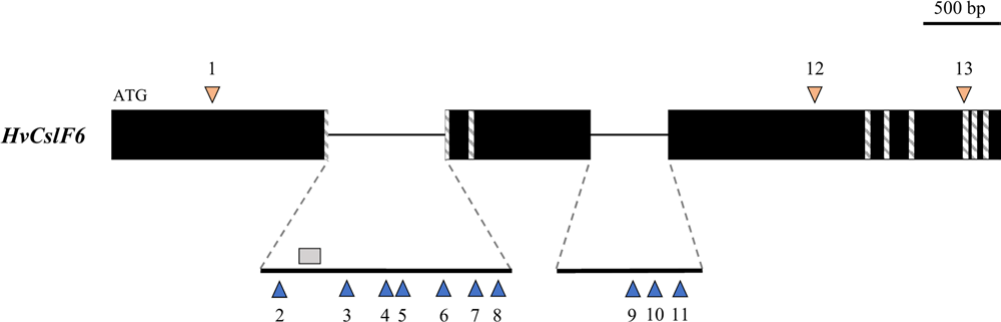
*HvCslF6* gene structure and relative position of the 13 SNPs identified across 1,336 barley accessions from the exome capture dataset. *HvCslF6* coding regions are shown in black boxes and transmembrane domains in stripe rectangles. Triangles represent SNPs (orange within CDS and blue within introns) and the grey box indicates the location of BdTHX1 binding site^53^.

An exome capture sequencing dataset comprising 1,336 barley accessions (including wild, landrace, and elite barley) was used to survey *HvCslF6* for sequence variation. Three polymorphisms (MAF < 5% cut off applied) were found within the *HvCslF6* coding region: two synonymous SNPs (first and third exon) and a non-synonymous SNPs on the third exon A590T, also described by Taketa *et al*.^29^. 10 SNPs were identified in non-coding regions of *HvCslF6*, seven were located within the first intron and three towards the end of the second intron (Fig. 3). Based on the predicted *HvCslF6* gene structure, detected SNPs within introns are not expected to affect splice junctions. Overall, the 13 SNPs found within *HvCslF6* had a low frequency across the genotypes analysed (Table 2); alternate alleles were present in approximately 19% of the barley accessions included in this study except for two SNPs located in the first and second introns of *HvCslF6* (46.3% and 39.9%, respectively).

**Table 2:** Description of SNPs detected within *HvCslF6* from the exome capture dataset.

SNP ID	Physical position	Position within *HvCslF6*	Reference → alternate allele	# Samples with data in SNP	Major allele as genotype (%)	Minor allele as genotype (%)	Type of mutation
1	378,390,528	Exon 1	G → A	835	93.3	6.0	Synonymous (aa 58)
2	378,390,290	Intron 1	C → A	725	94.2	5.1	
3	378,390,144	Intron 1	G → A	190	83.7	16.3	
4	378,389,638	Intron 1	T → C	65	83.1	16.9	
5	378,389,540	Intron 1	G → A	65	86.2	13.9	
6	378,389,490	Intron 1	G → A	81	81.5	18.5	
7	378,389,304	Intron 1	G → A	99	87.9	12.1	
8	378,389,172	Intron 1	G → A	136	52.9	46.3	
9	378,387,632	Intron 2	A → G	241	66.8	32.8	
10	378,387,538	Intron 2	T → C	173	60.1	39.9	
11	378,387,440	Intron 2	G → A	337	64.4	35.3	
12	378,386,503	Exon 3	G → A	887	92.6	6.7	Non-synonymous (A590T)
13	378,385,766	Exon 3	T → C	1323	93.0	7.0	Synonymous (aa 835)

### Limited sequence variation in a −3,000 bp *HvCslF6* putative promoter region

**Figure 4:**
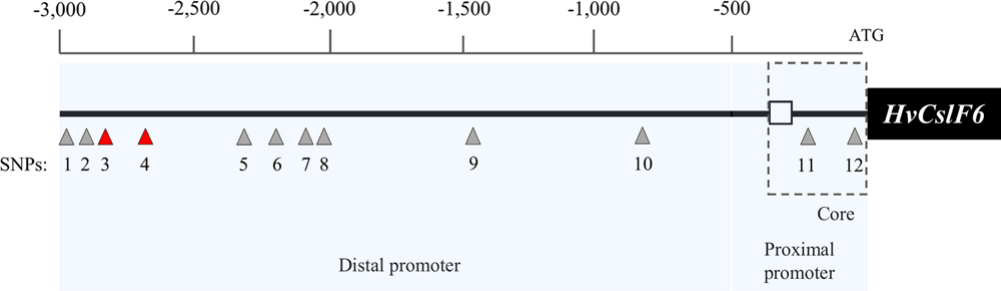
*HvCslF6* promoter structure representation containing 11 SNPs identified across a subset of 35 barley accessions. Previously described SNPs^15^are indicated by red triangles whereas unique SNPs identified in the current study are shown in grey. Consensus TATA box motif [TATAWAWN^70^] is represented by a white box. Distal, proximal and core promoter regions^71^ are shown as shaded boxes.

We identified 12 SNPs across the 3,000 bp promoter region of 35 barley genotypes described in Table S2. Two of these SNPs were located in the proximal promoter (defined as −500 bp upstream the start codon), which is generally known to contain key *cis*-acting regulatory elements in plant species^38^, while the remaining polymorphisms were detected at least −500 bp upstream of the *HvCslF6* start codon (Fig. 4). No correlation was found between the 12 SNPs identified within the region upstream of the *HvCslF6* start codon and the natural variation in grain (1,3;1,4)-β-glucan content in the germplasm analysed (a subset of 25 elite barley cvs split in two groups with contrasting grain (1,3;1,4)-β-glucan content, 6.3% and 3.0% (w/w) respectively, Table S2). The *HvCslF6* promoter sequences of *Hordeum vulgare* subsp. *spontaneum* ‘Caesarea’ and progeny lines, OSU105 and OSU127 contained six SNPs (five of them located in the distal and one in the proximal promoter region) that were unique compared with the other genotypes analysed (Table 3). Two of these were present in a set of 12 polymorphisms previously reported in six Australian barley lines^15^. Both single nucleotide substitutions were detected in the distal promoter.

**Table 3:** SNP location and details detected in a −3,000 bp *HvCslF6* upstream region across 35 barley accessions. SNP ID matches their representation on Fig. 4 from left to right. cv Morex was used as the reference sequence for SNP identification^51^.

SNP ID	Location (bp)	Reference → alternate allele	Genotype	Description
1	−2,798	T → G	Bowman, BW837	Current study
2	−2,655	C → T	Caesarea, OSU105, OSU127	Current study
3	−2,629	C → T	Caesarea, OSU127	Wong *et al*., (2015); Current study
4	−2,349	A → T	Caesarea, OSU105, OSU127	Wong *et al*., (2015); Current study
5	−2,338	G → A	Bowman, BW837, BW638, BW835, Glacier, Caesarea, OSU105, OSU127	Current study
6	−2,294	A → G	Caesarea, OSU105, OSU127	Current study
7	2,067	A → G	Caesarea, OSU105, OSU127	Current study
8	2,017	T → G	Egmont	Current study
9	−1,490	insC	Egmont	Current study
10	−777	A → C	Glacier	Current study
11	−262	C → A	Caesarea, OSU105	Current study
12	−59	T → G	Caesarea	Current study

## Discussion

Quantification of (1,3;1,4)-β-glucan content during late endosperm differentiation (15 DPA) until grain maturity (38 DPA) revealed two main groups with divergent grain (1,3;1,4)-β-glucan content. (1,3;1,4)-β-Glucan quantification was not assessed during early grain development stages (3–5 and 8–10 DPA) based on previous data that suggested levels increase later during grain development^1,39^ as well as to avoid potential errors in determining small concentrations of (1,3;1,4)-β-glucan. Based on *HvCslF6* CDS variation, the only non-synonymous SNP (A590T) identified in the exome capture dataset is unlikely to explain differences in grain (1,3;1,4)-β-glucan content^5,29,30^. This suggests that another aspect of *HvCslF6* biology, possibly transcript abundance, or the transcript abundance of other genes potentially contributes to (1,3;1,4)-β-glucan content. Previous QTL studies across different barley populations^37,40–43^ identified several genomic regions (1 H, 2 H, 3 H, 5 H and 7 H mainly) collocating with genes putatively involved in the synthesis, remodelling or degradation of (1,3;1,4)-β-glucan which could contribute to the variation in grain (1,3;1,4)-β-glucan content. A QTL interval on 1 H identified in these studies contained *HvCslF9*, a member of the same gene family as *HvCslF6*, and *HvGlbI* which is capable of hydrolysing (1,3;1,4)-β-glucan. Therefore, we quantified wholegrain transcript abundance of *HvCslF6*, *HvCslF9*, and *HvGlbI* in addition to (1,3;1,4)-β-glucan content. The use of wholegrain samples rather than isolated endosperm was important since this reflects the end use of barley grain in malting and feed applications. Despite this, previous studies^4,13^ on isolated endosperm tissue are reflective of our wholegrain development series for the targeted genes.

The dose-dependent role of *HvCslF6* in (1,3;1,4)-β-glucan biosynthesis has previously been demonstrated in barley, where endosperm-specific over-expression led to a considerable increase in (1,3;1,4)-β-glucan concentration^12^. In addition, the functional characterization of *HvCslF6* orthologs confirmed a similar role in wheat^44^ and rice^45^. In the present study, *HvCslF6* and *HvGlbI* mRNA abundance was examined in four elite barley cvs with contrasting grain (1,3;1,4)-β-glucan to understand if the combination of transcript abundance of genes encoding biosynthetic and hydrolytic enzymes could explain mature grain (1,3;1,4)-β-glucan content. Although transcript levels and profile varied between the cvs during grain development, the combined variation did correlate with mature grain (1,3;1,4)-β-glucan levels for some cvs, but not for others. It has been observed previously that just considering the expression profile of *HvCslF6* does not describe grain (1,3;1,4)-β-glucan content in Australian cvs Sloop and Himalaya^13^, and more recently in cvs CDC Bold, Beka, Logan, Harrington and breeding lines ‘TR251’, ‘TR306’^15^. However, qRT-PCR showed that for some cultivars (Dew and Egmont) at least a balance between biosynthetic and hydrolytic transcript abundance, and hence activities, likely impacts final (1,3;1,4)-β-glucan content. For others (Imidis and Gull) the lack of a direct relationship between transcript abundance of *HvCslF6* and *HvGlbI*, and grain (1,3;1,4)-β-glucan content raises several possibilities. One, that there may be other genes contributing to final grain (1,3;1,4)-β-glucan content, and two, that wholegrain transcript abundance may not necessarily correlate with protein amount and enzyme activity. Although the lack of polymorphisms in the *HvCslF6* coding sequence (see below) argues against any cultivar-specific modification to enzyme activity, it is possible that in some cultivars, polymorphisms in non-coding regions could impact interactions with upstream transcription factors and this could contribute to variation in transcript abundance and grain (1,3;1,4)-β-glucan.

Based on the current grain development series, 2-row genotypes (cvs Dew, Imidis, Egmont, Gull) had highest levels of *HvCslF6* expression at 8–10 DPA and 15 DPA during grain development. Similarly Nemeth *et al*.^44^ observed that the wheat *CslF6* ortholog*, TaCslF6*, was highly expressed during mid-endosperm development in cv Hereward, corresponding to the grain filling stage, showing maximum expression levels from 8–15 DPA. In a more recent study, the average *TaCslF6* mRNA levels across 10 wheat cvs were abundant at 21 and 28 DPA although different expression patterns were found within this subset of wheat genotypes, showing significant differences in *TaCslF6* transcript abundance at 21, 28 and 40 DPA^46^. These expression profiles are consistent with a function of (1,3;1,4)-β-glucan as a destination for energy storage in the form of metabolizable glucose during grain filling and germination^47,48^. As mentioned in Wong *et al*.^15^, differences in *HvCslF6* expression levels during late grain development might impact (1,3;1,4)-β-glucan accumulation in mature barley grain. In our experiment cv Egmont [6.09% (w/w) ± 0.19] showed an increase in *HvCslF6* expression at 38 DPA and no *HvGlbI* expression at this time point in contrast to low grain (1,3;1,4)-β-glucan cvs Dew [4.13% (w/w) ± 0.25] and Imidis [4.26% (w/w) ± 0.14]. Additionally, cv Egmont showed a 14.10% increase in (1,3;1,4)-β-glucan content from 32 DPA and 38 DPA coupled with an increase in *HvCslF6* expression, but a lack of *HvGlbI*.

In the cvs included in the present study the transcriptional profile of *HvGlbI* has a peak in expression at 15 DPA and 24–26 DPA, suggesting a potential role for this enzyme in determining (1,3;1,4)-β-glucan content during the later stages of grain development. A particularly high level of *HvGlbI* expression was observed at 15 DPA in cv Dew, which is characterized by a relatively low (1,3;1,4)-β-glucan content in mature grain compared to cvs Egmont and Gull (both with higher (1,3;1,4)-β-glucan content). In contrast, for other genotypes we observed concurrent high or low *HvCslF6* and *HvGlbI* transcript, which could initially appear to be counterintuitive. Concurrent synthase and hydrolase activity may contribute to re-modelling of the nascent polysaccharide, to hydrolysis of the polysaccharide off the synthase, or provide a rapidly degradable source of glucose in the developing grain due to its unbranched structure compared to starch^20,47,49^. Additionally, potential expression differences in (1,3;1,4)-β-endoglucanase *HvGlbII*, other putative β-glucan glucohydrolases (*HvExoI* and *HvExoII*) or β-glucosidases that were not investigated in this study may also contribute to variation in grain (1,3;1,4)-β-glucan content. Recently, Betts *et al*.^28^ reported expression differences of (1,3;1,4)-β-endoglucanases (*HvGlbI* and *HvGlbII*) in simulated malting conditions across malting and feed barley cvs. *HvGlbI* expression was three-fold higher than *HvGlbII* whose expression is known to be restricted to the aleurone under GA regulation^26^. However, since a direct relationship between transcript abundance and activity levels cannot be assumed, the quantification of (1,3;1,4)-β-glucanase activity in future studies could provide useful data towards understanding variation during grain development in the cultivars investigated here. Alternatively, this could indicate that the relationship between *HvGlbI* expression, isoenzyme EI activity, and consequently (1,3;1,4)-β-glucan content is nonlinear. It is possible that *HvGlbI* acts in combination with other remodelling and degrading enzyme partners to explain differences in grain (1,3;1,4)-β-glucan content.

Based on the grain development series used in the current study, the expression of *HvCslF9* was predominantly detected from 3–5 DPA, except for a later expression peak in cv Gull (8–10 DPA). Burton *et al*.^13^ also observed a peak of expression for *HvCslF9* for cv Sloop at 8 DAP, while at other time points in both genotypes included in their study *HvCslF9* transcripts were absent or at extremely low levels. Similarly, in the current study during mid and late grain development, and coinciding with endosperm differentiation and maturation, *HvCslF9* expression was almost undetectable across all genotypes. While other independent *HvCslF9* expression studies support a peak expression of this gene during endosperm differentiation stages^13^, *HvCslF9* over-expression did not increase (1,3;1,4)-β-glucan content in transgenic barley grain^12^, with similar results obtained in wheat addition lines^50^. The fact that the genomic location of *HvCslF9* and *HvGlbI* [1 H, 48.1 cM and 1 H, 54.4 cM, respectively^51^] co-locates with association peaks based on two independent QTL and genome-wide association studies (GWAS) on grain (1,3;1,4)-β-glucan content^5,37^ might suggest that variation in (1,3;1,4)-β-glucan is due to *HvGlbI* (1,3;1,4)-β-glucanase activity and not *HvCslF9* biosynthetic activity.

In the current study, a survey of *HvCslF6* sequence variation in 1,336 exome-captured barley accessions identified three SNPs within the *HvCslF6* coding sequence, two synonymous and one non-synonymous SNP, A590T which doesn’t explain differences in grain (1,3;1,4)-β-glucan content^5,15,29,30^. Based on the model of HvCSLF6 described by Schwerdt *et al*.^52^, the A590T substitution is proximal to a 55 amino acid insert that is specific to CSLF6 proteins of the grasses and absent in other CSLF proteins. While several amino acids under positive selection (non-synonymous to synonymous nucleotide substitution rate greater than 1) were described flanking the 55 amino acid insert and at other predicted transmembrane domains, low levels of sequence variation were found in CSLF6 unlike other CSLF proteins in grasses. The role of the CSLF6-specific amino acid insert, far from the active site, is not entirely clear however it contains charged aa residues and a conserved Cys residue which might facilitate the interaction with other protein/s^12,52^.

In the same exome-captured dataset, 10 SNPs were detected in non-coding regions of *HvCslF6*, mostly within the first intron, confirming that natural variation within this gene is rare. Recent work in *Brachypodium distachyon* identified a trihelix family transcription factor (BdTHX1) which binds to a GT-motif in the second intron of *BdCslF6*^53^. The same motif is found in the first intron of *HvCslF6* and another putative GT-motif is present in the second intron. However, the 10 SNPs within *HvCslF6* introns identified in this study did not affect the THX1 binding site or GT-motifs described by Fan *et al*.^53^.

Similar to the intronic regions of *HvCslF6*, low levels of variation were identified in the putative *HvCslF6* promoter sequence. In a −3,000 bp region upstream of the *HvCslF6* start codon, DNA sequence alignments identified only 12 SNPs across 35 barley accessions representing natural (1,3;1,4)-β-glucan variation. Polymorphisms were mostly found in the *HvCslF6* distal promoter, in which two SNPs were found in common with a set of six Australian cultivars previously analysed for sequence variation in 5’ and 3’ UTR regions^15^. These data indicate that there is remarkably narrow variation within *HvCslF6* promoter and enzyme coding sequences. None of these SNPs appeared to associate with variation in (1,3;1,4)-β-glucan content. Although genetic diversity will likely be lower when considering elite cultivars, Russell *et al*.^54^ observed 745,815 SNPs in high confidence exons across 20,729 high confidence gene models, representing an average of 35.9 SNPs per gene model in a collection of 267 wild and landrace barleys. The low sequence diversity in *HvCslF6* identified in our current study and by others^15,30,31^ appears to highlight atypical conservation of the *HvCslF6* nucleotide sequence, possibly due to its indispensable role in (1,3;1,4)-β-glucan synthesis and the importance of this polysaccharide in the grasses, or a common origin of the gene^55^. This role has been confirmed via the characterization of barley chemically-induced mutants that completely lack grain (1,3;1,4)-β-glucan and exhibit poor agronomic traits^29,56^, or partially functional *HvCslF6* mutants with less severe phenotypic effects^31^. Thus far, evidence suggests that the basis for differences in grain (1,3;1,4)-β-glucan is not linked to any polymorphism within *HvCslF6* and hence other regulatory sequences or proteins, acting independently or in combination, are likely to be involved.

Mature grain characteristics, including size and shape, were not obviously correlated with the amount of grain (1,3;1,4)-β-glucan or expression differences in genes that produce enzymes that synthesize or hydrolyze (1,3;1,4)-β-glucan. Hence, natural diversity for barley grain size seems unlikely to affect (1,3;1,4)-β-glucan concentration. This is despite recent work on the barley *lys3a* shrunken endosperm mutant showing that defects in hordein synthesis could potentially affect *HvCslF6* transcript abundance^57,58^. Shrunken endosperm barley mutants typically exhibit defects in starch biosynthesis and embryo development, but the (1,3;1,4)-β-glucan content was not quantified^59^. Since (1,3;1,4)-β-glucan has been proposed to act as an alternative source of stored glucose^60^, knowing the (1,3;1,4)-β-glucan content of shrunken endosperm mutants may help to explain the role of this polysaccharide and the regulation of *HvCslF6*. Furthermore, by comparing *Brachypodium* and barley grain during development Trafford *et al*.^61^ suggest that starch synthesis influences endosperm cell enlargement and as a consequence endosperm size. Therefore, although the relationship between starch and grain size is well understood, perhaps a wider screen of mutant germplasm^62^ might reveal a link between grain size and shape, and (1,3;1,4)-β-glucan content.

## Material and Methods

### Plant material for grain development series

Four elite 2-row spring *cv*s: Dew, Imidis, Egmont and Gull were used in this experiment. Based on previous work^5^, these *cv*s represent phenotypic extremes of grain (1,3;1,4)-β-glucan concentration (2.1–6.7% w/w). Three biological replicates across six grain development stages: 3–5, 8–10, 15, 24–26, 32 and 38 days post anthesis (DPA) were collected from material grown in glasshouse conditions of 16 h light/8 h dark, until maturity at The James Hutton Institute, United Kingdom (Feb–May 2015). In the present grain development series, DPA were considered equivalent to days after pollination (DAP), which has been used in previous studies^4,13,15^. For each genotype, six developing grains from the central part of the spike were collected at each developmental stage and snap frozen in liquid nitrogen for further mRNA and (1,3;1,4)-β-glucan quantification analyses.

### Isolation of mRNA and cDNA synthesis

For mRNA isolation, we collected three biological replicates from the time points described above, and for each time point included three technical replicates. Samples were ground into a fine powder (100 mg) in liquid nitrogen and mixed with 1 mL TRIzol (Thermo Fisher Scientific, Waltham, USA). Manufacturer’s instructions were followed with several modifications: (1) After phase separation, supernatant was transferred to a fresh tube and mixed with 0.25 mL isopropanol followed by 0.25 mL 0.8 M sodium citrate/1.2 M sodium chloride (per 1 mL TRIzol used, both) to help removal of polysaccharides. Samples were incubated for 10 min, at room temperature. (2) After an ethanol wash (1 mL 75% ethanol per 1 ml of TRIzol used), RNA pellets were re-suspended in 400 µL RNase-free water on ice. (2) For RNA purification, 400 µL chloroform/isoamyl alcohol (Sigma Aldrich, St. Louis, USA) was added, mixed well and centrifuged (16,000 × g, 4 °C) for 10 min. The top phase (~300 µL) was removed to a clean 1.5 mL Eppendorf tube on ice and 100 µL RNase-free water was added to the original tubes, mixed well and centrifuged (same conditions) for 5 min. The top phase (~100 µL) was removed and added to the 300 µL already collected in tubes, remaining on ice. (3) For RNA precipitation, samples were mixed with 1 mL absolute ethanol and 40 µL 3 M sodium acetate and stored at −80 °C overnight. The next day, samples were centrifuged (16,000 × g, 4 °C) for 10 min and supernatants were removed. RNA pellets were washed in 1 mL 70% ethanol and re-suspended in 100 µL RNase-free water. cDNA synthesis (1 µg total RNA) was performed using the RNA to cDNA EcoDry™ Premix (Takara, Kyoto, Japan) according to the manufacturer’s instructions.

### qRT-PCR

Quantitative real-time PCR (qRT-PCR) was performed in a StepOne Real-Time PCR machine (Thermo Fisher Scientific, Waltham, USA) using PowerUp SYBR Green Master Mix (Thermo Fisher Scientific, Waltham, USA). Three replicate qRT-PCR reactions were performed for each cDNA sample including three negative controls using RNAse-free water. Gene specific primers and qRT-PCR reaction conditions were used as described in previous studies^12,13^. Absolute mRNA quantification of *HvCslF6, HvCslF9* and HvGlbI was performed using three housekeeping genes for normalization: *α-tubulin, (α-tub) glyceraldehyde 3-phosphate dehydrogenase (GAPDH)* and *heat shock protein* (*HSP70*) (Table S3). Multiple control gene normalization was performed as described in Vandesompele *et al*.^63^. Normalized mRNA copies were calculated based on standard concentrations. Each gene standard (10^1^–10^7^ mRNA copies/µL) for *HvCslF6*, *HvCslF9*, *HvGlbI*, *α-tubulin*, *GAPDH* and *HSP70* was generated by HPLC at The University of Adelaide^13^.

### Quantification of (1,3;1,4)-β-glucan in developing grain

Grain (1,3;1,4)-β-glucan levels were determined by a modified version of the Megazyme β-Glucan (Mixed Linkage) Assay Kit (Megazyme Int., Wicklow, Ireland)^12,64^ which permits the analysis of small samples (15 mg). Each biological replicate consisted of three barley grain per genotype and time point. Barley grain samples were weighed before and after a 72-h incubation at 65 °C to calculate dry mass and milled in a Powerlyser tissue homogenizer (MO BIO, CA, USA). Three independent barley flour samples (3 × 15 mg samples) were obtained and averaged for final grain (1,3;1,4)-β-glucan quantification, calculated as % of dry weight (w/w). Two technical replicates were performed on all samples using the Megazyme kit apart from a standardized barley flour control [4.05%–4.15% w/w of (1,3;1,4)-β-glucan, from Megazyme kit] included in each batch. Differences in (1,3;1,4)-β-glucan content for each developmental stage were determined by one-way ANOVA followed by Tukey’s honest significant difference (HSD) test using GenStat v19^65^.

### Phenotypic assessment of grain characteristics

A collection of 153 2-row elite barley lines was used for grain phenotypic measurements (Table S2). A sample of approximately 100–150 bulked seeds per genotype was used to measure seed weight, roundness, length, width, thickness and overall seed area using a SeedCount SC4 (Seed Count Australasia, Condell Park, Australia) at the University of Adelaide Barley Breeding Program/Laboratory, following manufacturer’s instructions. These data where combined with previously published grain (1,3;1,4)-β-glucan data^5^.

### *HvCslF6* promoter resequencing

In total, 35 barley genotypes were used to survey sequence variation within a −3,000 bp *HvCslF6* upstream region including: 25 elite barley cvs with divergent grain (1,3;1,4)-β-glucan content [2.1–6.7% w/w^5^], four ‘Bowman’ Near Isogenic Lines (NILs): BW840, BW837, BW638 and BW835^62^ with introgressions spanning the region where *HvCslF6* maps to chromosome 7 H, two Recombinant Chromosome Substitution Lines (RCSLs), OSU105 and OSU127^66^, and their corresponding parental lines (Table S2). RCSL genotypes were chosen based on their introgressions from cv Caesarea (*Hordeum vulgare* subsp. *spontaneum*), which cover the genomic location of *HvCslF6*. Genomic DNA was isolated from seedlings using the Qiagen DNeasy Plant Mini Kit (Qiagen, Hilden, Germany) according to the manufacturer’s instructions. PCR amplification of a −3,000 bp *HvCslF6* upstream region was achieved by dividing the fragment into four overlapping PCR reactions (Table S4). PCR cycle details are as follows: 98 °C for 2 min; 5 cycles of 98 °C for 15 s, 60 °C for 20 s, 72 °C for 1 min with a touchdown on the primer annealing step of −1 °C/cycle; then 35 cycles of 98 °C for 15 s, 56 °C for 20 s, 72 °C for 1 min; followed by 72 °C for 2 min. PCR amplicons were purified using ExoStar™ (GE Healthcare UK Ltd., Buckinghamshire, UK) following the manufacturer’s instructions. Sanger Sequencing was performed using an ABI3730 DNA Analyzer (Applied Biosystems Inc., Foster City, USA) at The James Hutton Institute. Resulting sequences were aligned to cv Morex *HvCslF6* reference sequence retrieved from the barley genome explorer, Barlex^51^ and analysed with Geneious V.9^67^ to identify polymorphisms.

### Exome capture dataset

The genomic position of *HvCslF6* (HORVU7Hr1G070010.3) on the physical map was retrieved using the Barlex database^51^, available at: http://www.barlex.barleysequence.org. Sequence variation of *HvCslF6* was analysed in 1,336 exome sequenced barley accessions (Unpublished, The James Hutton Institute). This dataset comprises a georeferenced collection of exotic barley alleles including 340 landraces (*Hordeum vulgare* ssp. *vulgare*), the Spanish core collection^68^, 288 wild lines (*Hordeum vulgare* ssp. *spontaneum*) of which 80 correspond to the Barley1K collection from Israel^69^ and 2- and 6-row collections of contemporary European barley cultivars evaluated in previous projects (WHEALBI and CLIMBAR) at The James Hutton Institute. Polymorphisms identified in barley accessions with suspected heterozygosity and minimum allele frequency (MAF) of ≤5% (cut-off) were removed from the analysis as described in Russell *et al*.^54^.

## Supplementary information

Supplementary Material
