# Supplementary material for: Barley grain (1,3;1,4)-β-glucan content: effects of transcript and sequence variation in genes encoding the corresponding synthase and endohydrolase enzymes
Source: Sci Rep. 2019 Nov 21;9:17250. doi: 10.1038/s41598-019-53798-8 (PMC6872655; doi:10.1038/s41598-019-53798-8)

Table S1. Grain phenotypic measurements and (1,3;1,4)-β-glucan content in 153 2-row elite barley lines used for correlation analyses.

Accession	Average Weight	Roundness	Length Mean	Length StdDev	Width Mean	Width StdDev	Thickness Mean	Thickness StdDev	Avg Seed Area	(1,3;1,4)-β-glucan
Aapo	42.2	2.61	9.63	0.57	3.06	0.38	2.76	0.11	19.2	5.0
Acapella	52.2	3.17	9.9	0.73	3.43	0.53	2.21	1.18	22.6	-
Adonis	61.9	2.49	9.5	0.58	3.47	0.4	2.98	0.17	20.2	-
Agenda	16.7	2.61	9.15	0.68	3.08	0.5	2.55	1	18.2	4.4
Akita	64.3	2.42	9.6	3.35	3.27	1.09	2.95	0.01	24.1	-
Alabama	43.2	2.81	8.87	0.44	3.07	0.42	2.21	1.18	17.1	5.3
Alis	28.7	3.16	9.55	0.81	2.8	0.44	2.16	0.42	17.8	5.1
Alliot	47.6	3.07	9.41	0.55	3.21	0.43	2.35	0.87	19.5	5.3
Amourette	45.1	2.73	9.77	0.53	3.21	0.54	2.5	0.24	21.3	5.0
Anaconda	56.6	2.54	9.98	0.71	3.53	0.37	2.8	0.12	23	4.8
Annabell	47.4	2.6	9.14	0.67	3.54	0.39	2.66	0.71	21.3	4.2
Ardila	39	2.75	9.1	0.53	3.09	0.5	2.32	0.59	19.4	4.6
Astoria	46.8	2.62	9.68	0.71	3.31	0.39	2.56	0.13	21.8	4.0
Athena	49.4	2.34	9	1.34	3.38	0.56	2.85	0.18	20.6	4.6
Athos	36.2	2.62	9.8	0.92	3.22	0.43	2.67	0.04	21.4	5.0
Atlas	28.6	2.98	9.66	0.58	2.95	0.33	2.22	0.27	19.2	3.9
Auriga	27.3	2.66	10.08	0.56	3.28	0.51	2.66	0.07	21.6	4.3
Avec	39.6	2.92	9.65	0.53	3.26	0.37	2.24	0.64	20.7	4.6
Barke	33	2.34	9.14	0.35	3.13	0.54	2.85	1	20.6	4.3
Baronesse	39.7	2.79	9.65	0.78	2.92	0.49	2.43	0.43	19.3	-
Berac	38.4	3.15	8.92	3.25	3.11	1.03	1.93	0.96	21.2	4.5
Beryllium	51.6	3.64	9.78	0.62	3.49	0.47	2.02	1.27	24.1	4.5
Braemar	54.3	2.64	9.88	1.4	3.43	0.55	2.67	0.39	23.5	5.4
Brazil	27.3	2.88	9.45	0.63	2.79	0.39	2.33	0.29	18.6	4.4
Cabaret	34.9	2.65	9.9	0.42	3.44	0.52	2.65	0.05	21.9	4.9
Calico	56.1	2.76	10.19	0.67	3.42	0.58	2.59	0.57	23.9	4.2
Cambrinus	34.6	2.51	8.3	2.69	2.96	0.91	2.45	0.12	17.8	-
Camir	35	2.45	8.34	0.38	2.96	0.39	2.47	0.11	16.3	-
Campala	35.2	2.89	8.87	1.7	2.92	0.53	2.18	0.58	18	5.0
Casino	20.8	3.45	9.66	0.27	2.46	0.78	1.92	0.17	17.9	-
Catalina	54.5	2.43	8.51	4.1	2.97	1.25	2.66	0.08	24.3	5.2
Cecilia	44.8	2.73	9.32	1.55	3.21	0.55	2.35	0.54	21.6	5.4
Celebra	48.6	2.43	9.3	0.67	3.45	0.37	2.76	0.52	21.8	4.9
Cellar	44	2.89	10.1	0.79	3.21	0.31	2.32	0.54	21.7	6.0
Chariot	33.3	2.73	9.55	0.73	3.3	0.66	2.51	1	20.6	4.2
Charm	31	2.73	9.23	0.67	3.05	0.38	2.4	0.12	17.1	-
Chieftain	31.5	2.87	9.31	1.04	2.9	0.56	2.32	0.4	19.4	5.2
Chime	38.5	3	9.54	0.82	3.14	0.37	2.48	0.57	19.7	4.8
Clarity	48.3	2.45	9.28	0.64	3.36	0.31	2.73	0.16	20.6	4.7
Class	47.9	2.61	9.89	0.69	3.44	0.4	2.68	0.19	22.6	4.5
Cocktail	50	2.8	8.26	4.06	2.78	1.33	2.43	0.23	20.8	4.6
Colada	53.4	2.69	9.87	0.66	3.26	0.6	2.52	0.48	21.5	5.0

Cooper	47.4	2.55	9.5	0.86	3.1	0.53	2.7	0.06	18.8	-
Cribbage	37.8	2.52	9.56	0.6	3.26	0.44	2.77	1	20.5	4.8
Croydon	50	2.38	9.15	0.59	3.36	0.34	2.85	0.11	20.5	6.4
Crusader	36.2	4.62	10.19	1.02	3.08	0.59	1.81	1.07	20	5.3
Danuta	51.2	2.57	10.02	0.86	3.33	0.42	2.82	0.18	21.3	5.5
Delibes	43.7	2.78	9.43	1.43	3.03	0.49	2.49	0.3	20	4.2
Derkado	52.9	2.81	9.91	0.84	3.43	0.52	2.2	1.1	21.6	-
Draught	47.9	2.5	9.15	1.58	3.38	0.62	2.65	0.19	21.3	4.6
Drum	50.5	2.71	10.04	0.85	3.4	0.46	2.61	0.32	23.4	3.8
Elo	36.2	2.54	9.1	0.64	2.93	0.35	2.53	0.07	16.3	4.9
Extract	60	2.71	9.9	0.68	3.37	0.45	2.69	0.13	21	4.4
Fairytale	42.9	2.47	8.89	0.68	3.01	0.48	2.67	0.06	17	4.3
Felicie	43.1	2.66	9.45	1.2	3.17	0.52	2.54	0.35	20.7	5.2
Fleet	74.1	2.6	11.12	0.68	3.52	0.37	3.08	0.11	24.5	-
Formula	45.4	2.94	10.06	0.71	3.3	0.5	2.3	0.63	23.8	5.0
Forum	30.8	3.3	9.83	0.37	2.58	0.5	2.01	0.23	18.3	-
Foxtrot	55	2.58	10.15	0.73	3.37	0.39	2.96	0.19	21.6	4.8
Franklin	40	2.83	9.12	0.57	2.82	0.43	2.44	0.27	16.2	-
Gant	40.2	2.71	9.86	0.74	3.22	0.46	2.51	0.18	21.5	-
Global	43.9	2.61	8.85	0.7	3.41	0.38	2.57	0.65	19.7	4.8
Golden Promise	36.3	2.55	8.88	0.63	3.31	0.41	2.45	0.27	19.5	5.1
Goldie	37.9	2.48	8.98	0.69	3.36	0.73	2.55	0.2	20.7	-
Granta	54.3	2.69	10.63	0.71	3.42	0.47	2.78	0.14	24.5	4.3
Gull	38.5	2.35	8.12	3.66	2.79	1.05	2.61	0.01	20.3	-
Gundel	51.1	2.84	10.1	0.76	3.3	0.31	2.6	0.77	21.6	5.4
Hana	40	2.59	9.02	0.67	2.99	0.32	2.52	0.13	17	-
Harry	49.6	2.62	10.18	0.65	3.24	0.54	2.68	0.14	22.5	-
Hart	51.1	2.46	9.26	0.56	3.3	0.48	2.74	0.2	19.8	-
Hassan	45.1	2.47	9.31	1.45	3.45	0.54	2.64	0.19	21.8	5.3
Heather	33.3	2.45	8.69	0.22	3.05	0.45	2.7	1	17.4	4.9
Helmi	36	3	9.86	0.84	3.01	0.38	2.3	0.47	19.4	5.3
Heris	50.9	3.18	9.04	0.63	3.26	0.36	2.27	1	19.4	4.7
Heron	47.1	2.58	9.25	0.7	3.43	0.43	2.49	0.56	21.6	4.7
Hindmarsh	50	2.51	9.87	0.61	3.25	0.48	2.94	0.15	20.2	-
Hopper	49.2	2.7	10.1	0.76	3.43	0.42	2.6	0.15	22.9	4.9
Horizon	50	2.53	9.41	1.27	3.4	0.57	2.64	0.18	22.1	-
Host	40.3	2.5	8.72	1.97	3.12	0.67	2.58	0.13	19.4	4.6
Ida	44.6	2.94	10.46	0.59	3.42	0.57	2.31	0.69	23.3	5.3
Indola	39.1	2.76	9.03	0.69	3.08	0.36	2.3	0.83	18	4.8
Isabella	55	2.48	9.65	0.59	3.75	0.41	2.75	0.41	23.4	4.7
Jacinta	45.3	2.68	9.33	1.73	3.05	0.61	2.53	0.3	19.6	-
Jive	44.3	2.61	9.46	1.43	3.14	0.5	2.66	0.17	21.1	-
Keops	37.6	2.81	9.5	1.43	3.07	0.52	2.33	0.42	20.5	5.0
Klaxon	44.7	2.61	9.62	0.81	3.08	0.34	2.68	0.29	20	4.9
Kristaps	35	2.68	9.47	0.43	3.05	0.41	2.49	0.22	18.7	5.2
Laird	39.5	2.52	8.73	0.41	3.19	0.58	2.45	0.18	19.5	-
Lina	48.6	2.66	10.03	0.49	3.04	0.66	2.68	0.14	20.8	6.0

Linden	62.3	2.65	10.85	0.77	3.29	0.43	2.99	0.09	23.5	-
Lithium	45.2	2.68	9.8	0.73	3.36	0.44	2.53	0.28	22.7	3.9
Livet	31.1	2.87	9.31	0.61	2.7	0.46	2.4	0.34	16	5.3
Macarena	47.3	2.61	9.92	0.62	3.21	0.46	2.69	0.18	22	5.8
Macaw	55.4	2.43	10.15	0.44	3.54	0.45	3.02	0.04	23.3	-
Maris Mink	50.8	2.68	9.56	0.8	3.41	0.37	2.51	0.58	22	5.4
Maypole	52.1	2.78	10.38	0.84	3.37	0.42	2.58	0.5	23.6	4.6
Melitta	53.8	2.31	8.92	0.9	3.27	0.51	3.01	0.08	17.8	-
Midas	50.6	2.54	9.13	0.54	3.36	0.48	2.59	0.32	21	4.8
Mikado	41	2.73	9.53	0.7	2.9	0.42	2.52	0.2	18.3	4.5
Minstrel	47.4	2.42	9.22	0.74	3.43	0.37	2.73	0.24	20.5	4.7
Mundah	50	2.98	10.22	0.77	3.16	0.37	2.36	0.85	20.9	-
Neruda	46.2	2.66	9.99	0.78	3.39	0.36	2.62	0.44	23.2	5.2
Nimbus	28.5	3.19	10.13	0.8	2.74	0.45	2.14	0.31	18.8	3.8
Novello	45.5	2.62	8.2	3.5	2.85	1.15	2.51	0.37	20.8	4.6
Optic	30.8	7.96	9.12	3.42	2.51	0.88	1.81	1.15	18.1	-
Orbit	39.7	2.74	9.52	0.51	3.38	0.43	2.41	0.33	21.1	5.1
Paramount	44.8	2.92	10.7	0.64	3.4	0.41	2.52	0.2	24.4	4.4
Pewter	49.7	2.63	10.14	0.73	3.34	0.37	2.9	0.16	20.7	-
Pitcher	31.9	3	9.61	0.69	2.76	0.45	2.26	0.22	18.6	4.1
Poker	49	2.62	9.79	0.63	3.29	0.35	2.68	0.11	21.7	5.4
Polygena	20	2.58	7.89	1	2.97	0.38	2.35	1	17.4	4.6
Prisma	25	3.17	10.76	0.65	2.66	0.32	2.39	0.3	18.4	-
Prosa	25	3.17	10.76	0.65	2.66	0.32	2.39	0.3	18.4	-
Quartet	49.9	2.38	9.31	0.76	3.56	0.47	2.8	0.3	21.7	-
Quench	45	2.57	9.35	1.24	3.24	0.52	2.66	0.48	20.8	4.9
Ragtime	48.2	2.61	9.69	1.9	3.18	0.81	2.6	0.17	23.2	4.6
Rainbow	15.3	3.32	9.56	1.41	2.71	0.49	1.33	1.08	17.5	-
Rakaia	32.4	2.64	8.86	0.75	3.03	0.52	2.48	0.44	17.7	4.1
Rasa	42.8	2.65	9.38	0.62	3.31	0.47	2.53	0.4	20.8	5.4
Riviera	38.1	2.8	9.61	0.85	3.17	0.34	2.29	0.51	21.5	5.3
Roxana	34	2.85	8.89	2.05	2.83	0.61	2.24	0.3	18.7	4.1
Ruja	23.8	2.88	9.08	0.5	2.68	0.35	2.4	0.52	15.2	4.4
Saana	34.3	2.83	9.37	0.65	3.27	0.39	2.2	0.79	19.9	4.8
Sacha	48.6	2.79	10.89	0.59	3.38	0.57	2.72	0.19	23.7	4.3
Saloon	36.3	2.86	9	3.15	2.85	0.9	2.35	0.2	20.6	3.9
Scandium	46.8	2.55	9.91	0.66	3.27	0.38	2.73	0.17	21.3	4.0
Sebastian	51.2	2.54	9.26	1.32	3.4	0.51	2.7	0.47	21.6	4.9
Simba	46.7	2.62	9.84	0.67	3.57	0.47	2.63	0.58	23.3	4.9
Sj Christina	46.2	2.42	9.42	0.68	3.46	0.48	2.82	0.24	21.6	-
Skittle	50	2.52	9.68	0.83	3.39	0.37	2.75	0.53	21.6	3.7
Starlight	48.5	2.61	9.07	1.6	3.41	0.59	2.49	0.71	20.7	4.2
Static	43.2	2.62	9.69	0.9	3.34	0.4	2.55	0.33	21.7	5.6
Tabora	43.6	2.58	9.45	0.5	3.01	0.42	2.69	0.14	18.6	4.3
Tankard	47.3	2.43	8.9	0.63	3.42	0.36	2.66	0.28	19.9	5.0
Taphouse	48	2.59	9.35	2.79	3.1	0.8	2.74	0.34	21.9	4.3
Tartan	47.3	2.7	10.16	0.77	3.29	0.41	2.59	0.26	22.5	4.9

Thistle	47.1	2.67	9.65	0.97	2.9	0.35	2.52	0.17	17	5.1
Thrift	50	2.33	9.48	0.41	3.34	0.47	2.87	0.01	20	4.9
Toby	58.9	2.8	10.65	0.71	3.56	0.5	2.72	0.19	24.1	4.3
Toucan	48.9	2.58	9.76	1.37	3.37	0.62	2.68	0.41	22.7	5.0
Trinidad	44.4	2.69	9.09	0.56	2.9	0.38	2.32	0.76	16.5	-
Troon	49.4	2.64	9.82	0.83	3.3	0.32	2.7	0.19	20.4	4.6
Tucson	36.4	2.92	9.76	1.37	2.99	0.52	2.36	0.31	20.3	4.9
Tyne	38.1	2.93	9.48	0.54	2.98	0.34	2.31	0.27	19.2	5.4
Ursa	43.7	2.81	10.12	0.86	3.16	0.4	2.54	0.3	21.5	5.2
Vankkuri	38.9	3.34	11.33	0.6	2.8	0.38	2.28	0.28	21.9	-
Velvet	30	3.15	10.09	0.65	2.79	0.62	2.34	0.24	19.1	-
Viivi	37.3	2.85	9.83	0.9	2.93	0.48	2.49	0.09	19.9	6.0
Waggon	44.6	2.22	9.55	0.56	3.19	0.33	2.78	0.14	18.8	4.8
Weitor	42.3	2.68	9.52	0.82	3.11	0.5	2.57	0.39	19.7	-
Wicket	59.8	2.36	9.83	0.41	3.67	0.44	3.08	0.14	23.3	-
Widre	35.2	2.95	9.72	0.61	2.64	0.38	2.43	0.09	16.6	4.4
Wren	37.1	2.72	9.67	0.39	2.78	0.43	2.56	0.05	17.3	5.0

Table S2. Barley genotypes used for Sanger sequencing of a putative -3,000 bp *HvCslF6* promoter region. Grain (1,3;1,4)-β-glucan content for the first 25 accessions was determined by Houston *et al*., (2014). The asterisk (*), represents unknown (1,3;1,4)-β-glucan content in Bowman NILs, RCSLs (OSU lines) and parental genotypes.

Accession	(1,3;1,4)-β-glucan (% w/w)	Description
Egmont	6.7	High
Gull	6.6	High
Bulbur89	6.4	High
Brigitta	6.4	High
Croydon	6.4	High
Century	6.4	High
Harry	6.4	High
Tellus	6.2	High
Anni	6.2	High
Lenta	6.2	High
Mars	6.2	High
Isaria	6.1	High
Cellar	6.0	High
Kenia	6.0	High
Saloon	3.9	Low
Appaloosa	3.8	Low
Nimbus	3.8	Low
Dallas	3.7	Low
Skittle	3.7	Low
Volla	2.9	Low
Brewster	2.7	Low

Viskosa	2.3	Low
Dew	2.2	Low
Cork	2.2	Low
Imidis	2.1	Low
Glacier	*	Spontaneous mutant in a Sermo/7*Glacier (CIho 6976) line (7H)
BW840	*	Bowman NIL Collection
BW837	*	Bowman NIL Collection
BW638	*	Bowman NIL Collection
BW835	*	Bowman NIL Collection
Bowman	*	Bowman NIL Collection
Harrington	*	Recombinant Chromosome Substitution Line (RCSL); Parent
Caesarea	*	*Hordeum vulgare* subsp. *spontaneum;* RCSL Parent
OSU105	*	RCSL; 7H introgression
OSU127	*	RCSL; 7H introgression

Table S3. Linear regression parameters and goodness of fit (r^2) used for absolute mRNA quantification of *HvCslF6*, *HvCslF9* and *HvGlbI***.**

Standards	Slope (m)	Intercept (b)	Goodness of fit (r^2, standard curve)
α-tubulin	-3.71	33.97	1.86
GAPDH	-4.73	53.96	1.63
HSP70	-3.44	32.17	1.95
HvCslF6	-3.28	47.05	2.02
HvCslF9	-2.97	37.63	2.17
HvGlbI	-1.92	35.68	2.07

Table S4. Primer sequences used for the amplification of a -3,000 bp *HvCslF6* upstream region, divided in 4 PCR amplicons (~750 bp).

Name	Orientation	Primer sequence (5'–3')
1B_L	Forward	AGAGTATTGCTACGTACAACCAA
1B_R	Reverse	TCGTAAGCTGTACTGCAAAATAAA
2B_L	Forward	ACTATACGGCTCAACTTATATTGACCA
2B_R	Reverse	CGGAAGGAGGCTTGCCCTT
3B_L	Forward	AGTTCAGGGAGCTCCATCAG
3B_R	Reverse	CCTCCTCCACCGATTGATTG
4B_L	Forward	GCACAAGCTCACAAACCCC
4B_R	Reverse	GCTCATTGCTCCGCACGC

Figure S1. Phenotypic assessment of grain characteristics in cvs: Dew, Imidis, Egmont and Gull. a) Thousand grain weight, TGW, (g), b) grain surface area (mm^2), c) width (mm) and d) length (mm). Error bars represent standard error associated to three independent genotypes. Letters above each bar indicate significant differences across genotypes determined by one-way ANOVA (p-value <0.01 across all traits) followed by Tukey's multi-comparison test.

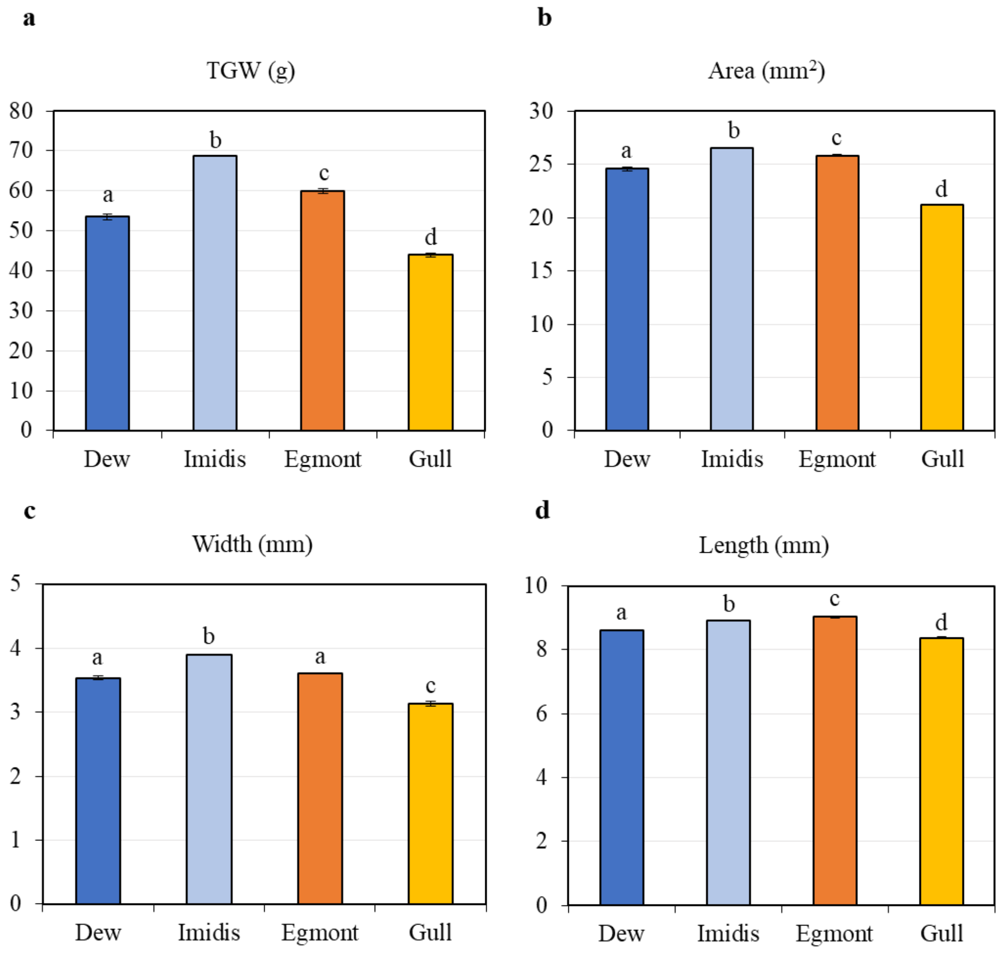

Supplement: Supplementary file 1 — Supplementary Material [file 41598_2019_53798_MOESM1_ESM.pdf]
